# Editorial: In celebration of women in signaling

**DOI:** 10.3389/fcell.2023.1326518

**Published:** 2023-11-14

**Authors:** Isabel Merida

**Affiliations:** Department of Immunology and Oncology, National Center for Biotechnology, Spanish Research Council (CSIC), Madrid, Spain

**Keywords:** PI3K, cancer, vesicles, integrin, bone loss

In spite of their elevated presence in the laboratories, women are still underrepresented in the top ranks of the scientific careers, including biomedical disciplines. This Research Topic offered a platform to promote the work of women scientists across the field of Signaling. Studies from researchers from Australia, Spain, United States and France have addressed a variety of issues that keep in common signaling alterations and human diseases.

Inappropriate activation of the PI3K signaling pathway lies at the core of most human cancers. Missense mutations of the PIK3CA oncogene are also found in affected tissues of a distinct set of congenital tumors and malformations collectively termed PIK3CA-related disorders (PRDs). Merechar et al. describe a new tool to test if gain-of-function Pik3ca mutations can cause vascular malformation syndromes. Expression of the most common post-zygotic activating mutation of Pik3ca in mice neural crest and related embryonic lineages allow the authors investigate the consequences of this mutation. Outcomes included macrocephaly, cleft secondary palate and more subtle skull anomalies. These murine phenotypes may aid discovery of new candidate human PRDs affecting craniofacial and vascular smooth muscle development as well as the reciprocal paracrine signaling mechanisms leading to tissue overgrowth.

Extracellular vesicles (EVs) secreted by cells have important functions in cellular homeostasis and cell-cell communication. Evolutionarily conserved from prokaryotes to eukaryotic cells, EVs play an important role in autocrine, paracrine, and endocrine signaling. Alterations in these mechanisms are implicated in multiple human disorders including prominent retinal degenerative diseases, like age related macular degeneration (AMD) and diabetic retinopathy (DR). Chatterjee and Sing review the current understanding of EVs in retinal (patho) physiology including disease-associated EV alterations in specific retinal diseases. Identifying different sub-populations of EVs in the retina in normal *versus* diseased condition(s), understanding the consequence of cell-specific EV cargo (e.g., DNA, RNA, protein, lipid) as well as understanding the mechanism behind the packaging of this cargo for retinal homeostasis would help to understand the mechanisms leading to retinal disease. The identification and characterization of cell specific EVs in body fluids like plasma, serum, VH, and aqueous humor in healthy subjects *versus* patients with retinal degenerative diseases will also be instrumental in development of biomarkers.

Signaling by integrins is crucial for normal epithelial tissue growth and development, and continues to be critical for tissue homeostasis and regeneration throughout life. Defects in the correct control of stem cells proliferation and differentiation can lead to abnormal tissue and is a hallmark of cancer. Rincón-Ortega et al. use the follicular epithelium (FE) of the *Drosophila* ovary as a model system to study the role of cell-ECM interactions in stem cell proliferation and differentiation during development. Their studies point to a role for the integrin-mediated cell-BM interactions in the control of epithelial cell division and subsequent differentiation.

An important aspect of signaling studies is that of investigate the potential effect side effects of medical treatments. Between 2005 and 2014, the prescription rate of antipsychotic use overall has increased in 10 out of 16 countries, and its use is associated with a number of side effects. A growing body of evidence suggests diminished bone mineral density (BMD) and increased fracture risk associated to antipsychotics. Weerasinghe et al. Review bone-associated signaling pathways and the direct impact of antipsychotics on different receptors present in bone cells.

